# Advances in risk predictive performance of pre-symptomatic type 1 diabetes via the multiplex Antibody-Detection-by-Agglutination-PCR assay

**DOI:** 10.3389/fendo.2024.1340436

**Published:** 2024-02-08

**Authors:** Devangkumar Tandel, Brigette Hinton, Felipe de Jesus Cortez, David Seftel, Peter Robinson, Cheng-ting Tsai

**Affiliations:** Research & Product Development, Enable Biosciences, South San Francisco, CA, United States

**Keywords:** type 1 diabetes mellitus, immunology, islet autoantibodies, pediatrics, autoimmune diseases

## Abstract

**Introduction:**

Achieving early diagnosis of pre-symptomatic type 1 diabetes is critical to reduce potentially life-threatening diabetic ketoacidosis (DKA) at symptom onset, link patients to FDA approved therapeutics that can delay disease progression and support novel interventional drugs development. The presence of two or more islet autoantibodies in pre-symptomatic type 1 diabetes patients indicates high-risk of progression to clinical manifestation.

**Method:**

Herein, we characterized the capability of multiplex ADAP assay to predict type 1 diabetes progression. We obtained retrospective coded sera from a cohort of 48 progressors and 44 non-progressors from the NIDDK DPT-1 study.

**Result:**

The multiplex ADAP assay and radiobinding assays had positive predictive value (PPV)/negative predictive value (NPV) of 68%/92% and 67%/66% respectively. The improved NPV stemmed from 12 progressors tested positive for multiple islet autoantibodies by multiplex ADAP assay but not by RBA. Furthermore, 6 out of these 12 patients tested positive for multiple islet autoantibodies by RBA in subsequent sampling events with a median delay of 2.8 years compared to multiplex ADAP assay.

**Discussion:**

In summary, multiplex ADAP assay could be an ideal tool for type 1 diabetes risk testing due to its sample-sparing nature (4µL), non-radioactiveness, compatibility with widely available real-time qPCR instruments and favorable risk prediction capability.

## Introduction

Type 1 diabetes (T1D) is a chronic autoimmune disease that affects more than 1.6 million children and adults in the US (1). Early detection of T1D is critical because the initiation of the autoimmune process that leads to T1D clinical presentation begins well in advance of the symptoms. Indeed, the American Diabetes Association (ADA), JDRF, and the American Endocrine Society published a joint statement in 2015 to recognize T1D as a disease continuum and update the definition of T1D diagnosis into several distinct stages (2–4). Patients with stage 1 and stage 2 T1D are positive for multiple islet autoantibodies and are at high risk of progressing to stage 3 T1D with clinical symptoms (e.g., hyperglycemia) (3, 4). This classification system was later confirmed by a joint statement from the NIDDK TrialNet study group (4).

Early diagnosis of stage 1 or 2 T1D with regular monitoring and follow-up could improve the clinical outcomes of T1D (5–7). First, the rates of diabetic ketoacidosis (DKA) at stage 3 T1D onset could be reduced, leading to lower HbA1c levels and a reduced risk of complications such as retinopathy and nephropathy (5–7). Second, FDA-approved therapeutics such as teplizumab could delay the clinical diagnosis of stage 3 T1D by years (8). Third, new generations of interventional therapeutics (e.g., NCT01773707 and NCT03428945) would benefit from a pool of early stage T1D patients to support ongoing clinical trials (9). This creates a positive feedback loop for T1D patients in that early diagnosis not only improves the outcome for the individual patient but also creates an opportunity to develop more effective therapeutics to benefit future T1D patients.

Nevertheless, the identification of stage 1 or stage 2 T1D patients is challenging because they are asymptomatic, and over 85% of them do not have a family history (2–4). Therefore, large-scale testing by the general public remains the only effective means of systematically identifying them. There are several methods to detect islet autoantibodies for the identification of patients with stage 1 or stage 2 T1D. The radiobinding assay (RBA) remains the gold standard and the most used assay format in large-scale testing programs for early T1D. Newer non-radioactive assays, such as ELISA, ECL, and LIPS, have been used either solely or in combination with RBA in recent testing programs (4, 10–13).

The multiplex Antibody Detection by Agglutination-PCR (ADAP) islet autoantibody assay used in this study was based on a highly sensitive ADAP platform (14–17). The multiplex ADAP assay is valuable for early T1D diagnosis because it uses a small-sample volume for testing (e.g., 1 µL–4 µL). Considering that a significant portion of stage 1 or stage 2 T1D patients are pediatric, reduction of sample collection burden with small volumes is critical. Furthermore, ADAP multiplexed all relevant islet autoantibodies in a single assay, further minimizing the sample volume requirement and increasing laboratory throughput. In addition, ADAP does not rely on radioactive reagents and uses standard RT-qPCR as an assay readout, making the test readily adoptable in standard clinical laboratories. These technical attributes and the high sensitivity/specificity of ADAP make it an attractive option for early T1D diagnosis.

Previously, this assay was validated for islet autoantibody detection in several studies with favorable performance characteristics, including the islet autoantibody standardization program (IASP) (10, 15–17). Nevertheless, these validations were conducted primarily on stage 3 new-onset or stage 4 established T1D patients. Despite satisfactory sensitivity and specificity, it was unclear whether the ADAP assay could be used to identify stage 1 or stage 2 T1D patients who are at risk of progressing to stage 3 T1D. Herein, we report the results of a pilot validation with retrospective serum samples from subjects who had been tested by RBA for islet autoantibodies and were followed up for 8 years. This unique cohort enabled the analysis of positive and negative predictive values (PPV and NPV) for T1D risk prediction, providing data to support the use of multiplex ADAP for the early diagnosis of presymptomatic T1D.

## Methods

### Human specimen characteristics

The specimens used in this study were obtained from the DPT-1 trial cohort sponsored by the NIDDK between 1994 and 2003 (18). Detailed patient recruitment and study protocols have been reported previously (18). Briefly, all participants were first- or second-degree of relatives of a person with T1D and were tested for islet cell autoantibodies (ICAs). Written informed consent was obtained from all the subjects in the study group. Patients with ICA autoantibodies were offered additional testing for GAD, IA-2, and insulin autoantibodies. Islet autoantibody testing records, follow-up records, and clinical diagnosis of stage 3 T1D records were available from the NIDDK biorepository.

Sera collected within 6 months of study enrollment were obtained from a total of 48 subjects who progressed to stage 3 T1D and 44 subjects who did not progress to stage 3 T1D during the follow-up. The subjects were randomly selected by the NIDDK central repository staff. These subjects either developed stage 3 T1D during follow-up or were followed up for at least 5 years. The demographic characteristics of the study participants are presented in **Table 1**. Notably, the study participants were predominantly non-Hispanic white individuals. There were more male than female participants. The samples were transferred to Enable Biosciences for multiplex ADAP analysis as de-identified-coded specimens. The result was only unblinded by the NIDDK central repository after testing was completed. The study was approved by the Western IRB (IRB number #20180015) to Enable Biosciences.

**Table 1:** Demographic of study subjects.

Subjects	Progressors	Non-progressors
Number	48	44
Age at testing (median and IQR) (year old)	8.1 (5.5–11.3)	13 (8.6–29.6)
Ethnicity	46 non-Hispanic white	42 non-Hispanic white
Sex	33 Male, 15 Female	26 Male, 18 Female
Age at stage 3 T1D diagnosis (median) (year old)	12.0 (9.5–14.8)	N/A

A detailed description of the study subjects was provided below.

### Multiplex ADAP assay analysis

Previously, we reported a multiplex ADAP method for detecting three islet autoantibodies (15). In addition, we described an automated Hamilton MicroLab STAR system to carry out the 3-plex ADAP assay (16). Recently, we expanded the assay to 5-plex to test for IAA, GADA, IA2A, ZnT8A, and TGA on a modified version of Hamilton MicroLab STAR to achieve full automation (17). Herein, we restricted the ADAP assay to a 4-plex assay to test for all four islet autoantibodies (IAA, GADA, IA2A, and ZnT8A) on the Hamilton MicroLab STAR system. Briefly, 4 μL of serum was incubated with 8 μL of DNA-barcoded autoantigens at 37°C for 30 min. If present in the specimens, autoantibodies agglutinate autoantigens into a dense immune complex. Then, 4 μL of the mixture was aspired and mixed with 116 μL of ligation mixtures, where nearby DNA in the dense immune complex was ligated to form a full-length DNA amplicon. Next, 25 μL of the above mixture was further mixed with 25 μL of PCR amplification mixtures containing primers for all five autoantibodies for a total of 13 PCR cycles using an on-deck thermocycler (ODTC, Inheco, Martinsried, Germany). The amplified products were then aspired to 384 well plates in which each well contained the cognate primer pairs for each autoantibody to achieve specific quantification by real-time quantitative PCR (RT-qPCR). The qPCR-ready plates were transferred to Bio-Rad CFX384 to enable an automated sample-to-answer solution. The samples were analyzed in a coded and randomized manner. The results were unblinded after sample testing was completed. The assay cutoffs were determined by testing 80 healthy controls and set at the 99th percentile. The cut-offs for IAA, GADA, IA2A, and ZnT8 were 0.99, 3.1, 2.3, and 2.0, respectively.

### Radiobinding assay analysis

The GAD, IA-2, and insulin autoantibody testing results were obtained from the NIDDK central repository database. Laboratory procedures for GAD, IA-2, and insulin autoantibody analyses have been extensively reported (18). Briefly, GAD and IA-2 autoantibodies were detetcted at the Barbara Davis Center (Denver, CO, USA). Insulin autoantibody levels were determined at the Barbara Davis Center or Joslin Diabetes Center (Boston, MA, USA). The cut-off values for the GAD and IA-2 assays were 0.032 and 0.049, respectively. For the insulin assays, the cut-off was 0.01 at the Barbara Davis Center and 0.02 at Joslin Diabetes Center. The cutoffs were determined using the 99th percentile of the healthy controls. A combined radiobinding assay was performed for GAD and IA-2 autoantibodies using radioactively labeled H3-GAD65 and S35-IA-2.

### Data analysis

Positive predictive value (PPV) was defined as the probability that a subject with a positive test result actually progressed to clinical presentation of the disease. The negative predictive value (NPV) was defined as the probability that a subject with a negative test result truly did not progress to disease clinical presentation. For instance, in this study, a positive test result was defined as having two or more islet autoantibodies, unless otherwise noted. The overall PPV was calculated based on the number of individuals that progressed to stage 3 T1D during the entire follow-up period, while the overall NPV was calculated based on the number of individuals who did not progress to stage 3 T1D during the entire follow-up period. The 5-year risk PPV and NPV were calculated similarly, except that we restricted the analysis to progression within 5 years. It should be noted that all study subjects had either been followed for 5 years or progressed to stage 3 T1D within 5 years. Kaplan–Meier estimates were used to plot progression risk and to compare probabilities of stage 3 T1D progression in subjects stratified by the number of islet autoantibodies, sex, or age groups. For all analyses, a 2-tailed P-value of 0.05 was considered significant. All statistical analyses were performed using Graphpad Prism (version 9.3.1).

### Data and resource availability

All data generated or analyzed during this study are included in the published article (and its online **Supplementary Files**). The reagents used in this study are available from the corresponding author upon request.

## Results

### Positive and negative predictive value of multiplex ADAP islet autoantibody assays

**Figure 1:**
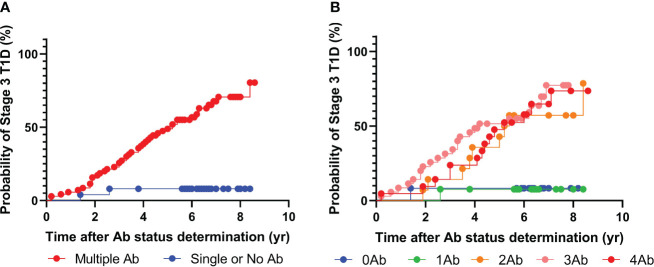
Progression to stage 3 T1D stratified based on islet autoantibody test results from 4-plex ADAP assay (GADA, IA2A, IAA, ZnT8). **(A)** Stratification based on harboring two or more islet autoantibodies. **(B)** Stratification based on number of islet autoantibodies.

In this study, we obtained 92 sera samples from 48 progressors and 44 non-progressors in the NIDDK DPT-1 study (18). All individuals either developed T1D during the follow-up period (progressors) or were followed up for at least 5 years (non-progressors). The sera were analyzed using multiplex ADAP assays for autoantibodies against GAD, IA-2, insulin, and ZnT8 (**Figure 1**, **Table 1**). Among them, 68 individuals tested positive for two or more islet autoantibodies, and 46 developed stage 3 T1D during the follow-up period. The median time from positivity for two or more islet autoantibodies to stage 3 T1D diagnosis was 4.2 years (Range: 1.0–8.4 years). Among the 24 individuals with one or fewer islet autoantibodies, only two individuals progressed to stage 3 T1D. One of them, diagnosed at the age of 13.1 years old, had a high level of GAD autoantibody and IA2 autoantibody level immediately below the cut-off, while the other, diagnosed at age of 27.8 years old, was negative for all islet autoantibodies. The overall positive predictive value (PPV) and negative predictive value (NPV) of the multiplex ADAP islet autoantibody assay based on the presence of two or more islet autoantibodies were 68% (46/68) and 92% (22/24), respectively. Alternatively, the PPV and NPV for progression to stage 3 T1D within 5 years of testing were 49% and 92%, respectively. The 5-year PPV was lower than the overall PPV because some individuals developed stage 3 T1D after 5-years of initial testing. The 5-year PPV observed in this study is consistent with that of other longitudinal follow-up studies (19–21).

Next, we sought to further explore whether individuals with two, three, or four islet autoantibodies would have distinct progression risks to stage 3 T1D (**Figure 1B**). Progression rates ranged from 64% to 70% (**Supplementary Table 1**). The median time from positivity for two or more islet autoantibodies to clinical presentation was 3.9, 3.1, and 4.4 years for individuals with two, three, and four islet autoantibodies, respectively.

Furthermore, an analysis was conducted to evaluate whether the types of islet autoantibodies would influence the risks of progression to stage 3 T1D (**Tables 2**, **3**). For individuals with two or more islet autoantibodies, if their autoantibody positivity included GAD, IA-2, insulin, or ZnT8 autoantibodies, the median time to diagnosis was 3.7, 3.8, 3.6, and 3.6 years, respectively, and the PPV were 68%, 68%, 65%, and 71%, respectively. If the autoantibody positivity included GAD/IA-2, GAD/insulin, GAD/ZnT8, IA-2/insulin, IA-2/ZnT8, Insulin/ZnT8 autoantibodies, the median time to diagnosis was 3.8, 3.6, 3.6, 3.8, 3.6, and 4.2 years, respectively and the PPV was 68%, 65%, 71%, 64%, 73%, and 67%, respectively.

**Table 2:** Multiplex ADAP islet autoantibody assay analysis results.

	Progressors (N = 48)	Non-progressors (N = 44)
Classification Scheme 1		
Two or more islet autoantibodies	46	22
One or less islet autoantibodies	2	22
Classification Scheme 2		
Four islet autoantibodies	14	7
Three islet autoantibodies	23	10
Two islet autoantibodies	9	5
One islet autoantibodies	1	11
Zero islet autoantibodies	1	11

In the classification scheme 1, subjects were classified based on whether they tested positive for two or more islet autoantibodies. In the classification scheme 2, subjects were classified based on the incremental number of islet autoantibody positivity.

**Table 3:** Impact of islet autoantibody pattern of progression to Stage 3 T1D.

Stage 1 or stage 2 T1D autoantibody positivity pattern	Median time to diagnosis	PPV
GADA	3.7	0.68
IA2A	3.8	0.68
IAA	3.6	0.65
ZnT8	3.6	0.71
GAD/IA2	3.8	0.68
GAD/IAA	3.6	0.65
GAD/ZnT8	3.6	0.71
IA2/IAA	3.8	0.64
IA2/ZnT8	3.6	0.73
IAA/ZnT8	4.2	0.67

For the 48 subjects tested positive for two or more islet autoantibodies by ADAP assays, additional analysis was conducted to evaluate impact of islet autoantibody pattern of progression risk. For GADA, IA2A, and IAA, these indicated the subjects were positive for two or more islet autoantibodies, and one of the islet autoantibodies was the specified autoantibodies. For GADA/IA2A, GADA/IAA, GADA/ZnT8A, IA2A/IAA, IA2A/ZnT8, and IAA/ZnT8, these indicated the subjects were positive for two or more islet autoantibodies, and two of the islet autoantibodies were the specified autoantibodies.

Therefore, the above observation indicated that the presence of multiple islet autoantibodies was a critical risk factor for progression to stage 3 T1D.

### Impact of age and sex on progression risk to stage 3 T1D

Patients positive for two or more islet autoantibodies might have distinct progression risks depending on their age and sex (22). To investigate this further, we first stratified the individuals into those under and above the age of 8 at the time of testing. For individuals under the age of 8 years, the multiplex ADAP assay had a PPV and NPV of 80% and 100%, respectively. For individuals over age of 8 years, the PPV and NPV were 58% and 90%, respectively. Therefore, the development of multiple islet autoantibodies at a young age appears to increase the risk of progression risk to stage 3 T1D. On the other hand, female patients had a slightly higher PPV than male patients (70% vs 67%), and the NPV was comparable (92% vs 91%).

### Impact of ZnT8 autoantibodies in prediction of T1D risk prediction

Recently, ZnT8 autoantibodies were discovered. The value of ZnT8 autoantibodies in aiding the diagnosis of new-onset clinical diabetes and risk predictions has been widely reported (23). It is of great interest to investigate whether the exclusion of ZnT8 autoantibodies would substantially impact the prediction of stage 3 T1D progression risk.

**Figure 2:**
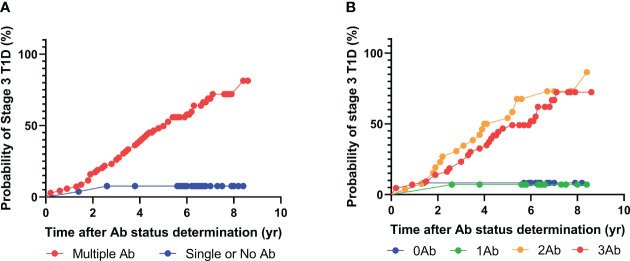
Progression to stage 3 T1D stratified based on islet autoantibody test results from 3-plex ADAP assay (GADA, IA2A, IAA). **(A)** Stratification based on harboring two or more islet autoantibodies. **(B)** Stratification based on number of islet autoantibodies.

To this end, the above analysis was performed again using only GAD, IA-2, and insulin autoantibodies (**Figure 2**). Intriguingly, 67 individuals tested positive for two or more islet autoantibodies, and 46 out of the 67 individuals eventually developed stage 3 T1D during follow-up. The median time from positivity for two or more islet autoantibodies to clinical presentations was 3.7 years. Similar to the previous analysis, only two out of 25 individuals with one or fewer islet autoantibodies progressed to stage 3 T1D. Accordingly, the positive predictive value (PPV) of the multiplex ADAP islet autoantibody assay with GAD, IA-2, and insulin autoantibodies was 68% (46/67) and the negative predictive value (NPV) was 92% (23/25). These predictive values were statistically indistinguishable from the predictive values when all four islet autoantibodies were included. The data thus support the use of three cardinal islet autoantibodies for the prediction of the risk of progression to stage 3 T1D.

### Comparison of predictive value to radiobinding assays

The prediction of the risk of progression to stage 3 T1D has been extensively studied in several landmark studies using radiobinding assays to measure islet autoantibodies. Indeed, the underlying DPT-1 study was one of the earliest nationwide longitudinal studies to provide critical insight into the natural history of T1D development and inspired and shaped study designs for many other recent studies. Importantly, radiobinding assay data from the DPT-1 studies were available from the NIDDK biorepository. We sought to compare the risk prediction between the multiplex ADAP assays and radiobinding assays. It should be noted that DPT-1 study was conducted between 1994 and 2003 (18). The design and protocols for radiobinding assays have been improved in recent studies (10). Nevertheless, the data will provide a valuable context to help understand whether the observed multiplex ADAP assay performance is satisfactory.

**Figure 3:**
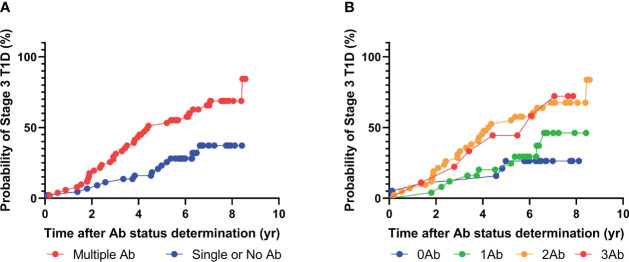
Progression to stage 3 T1D stratified based on islet autoantibody test results from radiobinding assays (GADA, IA2A, IAA). **(A)** Stratification based on harboring two or more islet autoantibodies. **(B)** Stratification based on number of islet autoantibodies.

Among the 92 patients with radiobinding assay data for GAD, IA-2, and insulin autoantibodies, 51 tested positive for two or more islet autoantibodies, and 34 progressed to stage 3 T1D during the follow-up period, with a median time to diagnosis of 3.4 years (**Figure 3**, **Table 4**). Fourteen of the 41 individuals with one or no islet autoantibodies progressed to stage 3 T1D, with a median time to diagnosis of 4.3 years. The overall PPV and NPV of the radiobinding assays were 67% and 66%, respectively. The 5-year PPV and NPV for the radiobinding assays were 51% and 78%, respectively.

**Table 4:** Radiobinding assay analysis results.

	Progressors (N = 48)	Non-progressors (N = 44)
Classification Scheme 1		
Two or more islet autoantibodies	34	17
One or less islet autoantibodies	14	27
Classification Scheme 2		
Three islet autoantibodies	10	3
Two islet autoantibodies	24	14
One islet autoantibodies	10	15
Zero islet autoantibodies	4	12

In the classification scheme 1, subjects were classified based on whether they tested positive for two or more islet autoantibodies. In the classification scheme 2, subjects were classified based on the incremental number of islet autoantibody positivity.

To compare performance, we first restricted the multiplex ADAP assay analysis to GAD, IA-2, and insulin autoantibodies, given that ZnT8 autoantibodies were not yet discovered at the time of the DPT-1 study. The multiplex ADAP assay and radiobinding assays had similar PPV of 68% and 67%, respectively. Nevertheless, the NPV differences were statistically significant (92% vs 66%, respectively). To elucidate the potential sources of the NPV differences, it was noted that multiplex ADAP assays identified 46 out of 48 patients that progressed to stage 3 T1D as multiple islet autoantibody-positive. In contrast, radiobinding assays only identified 34 out of 48 progressors as multiple islet autoantibody-positive, leading to a lower NPV.

We further compared the pattern of islet autoantibodies for the 12 progressors that had discrepant assigned risk profiles using multiplex ADAP and radiobinding assays (**Table 5**). Five of the 12 progressors were positive for GAD/IA-2/insulin autoantibodies, and the remaining seven individuals were positive for GAD/IA-2 autoantibodies with multiplex ADAP assays. On the other hand, seven out of the 12 progressors were single positive for GAD autoantibodies, one out of 12 was single positive for IA-2 autoantibodies, one out of 12 was single positive for insulin autoantibodies, and three out of 12 were negative for all islet autoantibodies by radiobinding assays. Thus, it appears that the discrepant risk profiles were not a result of specific islet autoantibodies. Nevertheless, it was noted that for the seven individuals with single GAD autoantibodies by radiobinding assays, their GAD autoantobody signals measured by ADAP ranged from 7.59 to 11.63, whereas those five were missed by radiobinding assays, and their GAD autoantibody signals measured by ADAP ranged from 4.79 to 6.54. Similarly, for the one individual with insulin autoantibodies by radiobinding assays, the ADAP signal was 4.92, while rest of 4 ADAP insulin autoantibody-positive individuals had signals from 1.07 to 2.07. These observations suggested that ADAP had improved sensitivities over radiobinding assays for GAD and insulin autoantibodies, as the samples were only radiobinding assay-positive if their ADAP signals were higher in values. In contrast, for IA-2 autoantibodies, the only radiobinding assay-positive sample had an ADAP signal of 7.78, but the remaining 11 samples had ADAP signals from 2.42 to 11.64. Should sensitivities be the only factor, we would expect those samples with ADAP signals above 7.78 to be positive by radiobinding assays. The fact that several samples with strong ADAP signals were negative by radiobinding assays implied that the two assays might have additional differences for IA-2 autoantibody detection, such as autoantibody epitopes and isotypes.

**Table 5:** Discordant results from subjects that eventually progressed to Stage 3 T1D.

Subject	ADAP			Radiobinding assay		
	GADA	IA2A	IAA	GADA	IA2A	IAA
Progressor 1	11.63	3.55	2.07	0.86	−0.03	0.00
Progressor 2	5.58	7.78	1.07	−0.03	0.74	0.00
Progressor 3	6.01	3.19	4.92	−0.05	−0.01	0.12
Progressor 4	6.55	2.42	1.52	0.01	−0.02	0.00
Progressor 5	6.01	11.64	1.83	−0.01	0.02	0.00
Progressor 6	11.21	3.68	0.65	0.86	0.01	0.00
Progressor 7	9.24	3.06	0.81	0.36	0.01	0.00
Progressor 8	10.50	8.49	0.70	0.43	0.01	0.00
Progressor 9	9.38	5.46	0.62	0.12	0.00	0.00
Progressor 10	7.59	8.39	−0.03	0.04	−0.03	−0.02
Progressor 11	9.27	4.60	0.18	0.19	0.02	0.00
Progressor 12	4.79	2.70	0.92	−0.04	−0.02	0.00

A total of 12 subjects that eventually progressed to stage 3 T1D within the following up period had discordant results by the multiplex ADAP assays and radiobinding assays. Given that radiobinding assays only analyzed GADA, IA2A, and IAA during the DPT-1 study, the ADAP data shown here were restricted to the same three autoantibodies. Positive results were highlighted in red.

Notably, the NIDDK biorepository had longitudinal radiobinding assay data for a portion of DPT-1 study samples. For these 12 progressors who were initially positive for one or fewer islet autoantibodies by radiobinding assays, five later developed two or more islet autoantibodies. The ADAP assay preceded the radiobinding assay by a median of 2.8 years for detecting two or more islet autoantibodies in these five samples. The remaining seven progressors did not develop two or more islet autoantibodies by radiobinding assays during the follow-up. While the sample size was limited, this is preliminary evidence that the ADAP assay could enable earlier diagnosis of stage 1 or stage 2 T1D.

The overall sensitivity of the multiplex ADAP sand radiobinding assay was 96% and 71%, respectively, whereas the overall specificity of the multiplex ADAP sand radiobinding assay was 50% and 61%, respectively.

## Discussion

Over the past two decades, our understanding of the risk factors, progression profiles, and prevention and intervention strategies for T1D has dramatically improved. Historically, T1D is a disease that can only be managed by insulin administration and glucose monitoring and cannot be prevented or cured. Teplizumab was recently approved by the FDA as the first drug to delay or prevent progression to stage 3 T1D (8). This has sparked widespread interest in building infrastructure to identify stage 1 or stage 2 T1D patients that may benefit from immunomodulatory drugs and create a pool of eligible patients to support the development of newer generations of interventional therapeutics (4). Considering that more than 85% of patients with stage 3 T1D have no family history, testing efforts have been increasingly directed toward the general population, including landmark Fr1da and ASK studies (24, 25).

The multiplex ADAP islet autoantibody assay may be a suitable tool for large-scale testing of stage 1 or stage 2 T1D in the general population. The ADAP assay features low sample volume consumption (as little as 1 µL–4 µL), is multiplex, and does not rely on hazardous radioactive reagents. These attributes are relevant in that most of the testing targets would be young children, where phlebotomy blood draw would create a substantial sample collection burden and decrease testing access. Extensive validation of the multiplex ADAP assay focused on evaluating assay performance in stage 3 or stage 4 T1D patients. While these validation data were promising in nature, they did not address the predictive value of T1D progression risk.

This study leveraged elegant retrospective samples from the DPT-1 study to fill this critical gap and provided valuable validation of risk prediction using the multiplex ADAP assay platform. The results showed satisfactory PPV and NPV values of 68% and 92%, respectively. Importantly, these data support the use of GAD, IA-2, and insulin autoantibodies to achieve effective risk prediction. In comparison, the radiobinding assays had PPV and NPV of 67% and 66%, respectively. The marked improvement in NPV was likely a combined result of the enhanced sensitivities of ADAP assays and intrinsic differences in assay epitope exposures. Notably, of the 48 individuals who eventually progressed to stage 3 T1D, the multiplex ADAP assay classified 46 as stage 1 or stage 2 T1D, whereas the radiobinding assay identified 34. These data complement previous validations using new onset/established T1D patient samples and demonstrate the robust performance of the multiplex ADAP assay.

Nevertheless, this study had some limitations. the DPT-1 study was conducted between 1994 and 2003. Therefore, the radiobinding assays used in the DPT-1 study improved over time. The observed lower performance of radiobinding assays in the DPT-1 study may not represent the performance of radiobinding assays (10). For instance, in a recent report in 2013 (21), radiobinding assays achieved an NPV of 87.3%–99.6% and a PPV of 61.6%–79.1%. These values were comparable to the multiplex ADAP assay performance reported in this study. Second, the sample size used in this study was limited. Third, the study was conducted using serum samples collected from phlebotomy blood samples. Finger-prick whole blood or dried blood spot should be used to fully realize the sample-sparing nature of the ADAP assay. Future studies should investigate risk prediction using ADAP assays with these easily collectable sample formats. Fourth, this study was primarily based on samples from relatives of T1D patients who tested positive by islet-cell antigen assays. It is desirable to conduct pilot testing with longitudinal follow-up in the general population setting to definitively evaluate the PPV and NPV. Finally, this study focused on clinical risk prediction accuracy and did not address the overall impact of improved prediction on patient outcomes and healthcare economics. Future studies should be designed to evaluate whether improved predictions can lead to better patient outcomes and economic savings.

In addition to radiobinding assays, several new generations of islet autoantibody assays have been developed and reported, including ELISA, electrochemiluminescence (ECL), and luciferase immunoprecipitation (LIPS) (10–13). It is desirable to compare the ADAP assay performance beyond the radiobinding assay with these newer assay formats. Based on the comparison results, it might be possible to design a T1D risk-testing algorithm in which a highly sensitive assay is used as the first-line screening assay and the sample is reflected in a confirmatory assay with a high positive predictive value. These types of algorithms may achieve performance above and beyond what is possible with a single assay format. Additional considerations should be considered when designing these algorithms. For instance, the first-line and confirmatory assays should be compatible with the same sample type. Furthermore, the first-line assay should have minimal sample consumption, such that sufficient samples are available for confirmatory assays. Meeting these requirements would prevent the need for additional sample collection and increase participation in testing.

In conclusion, this study provides valuable evidence for establishing the predictive value of the multiplex ADAP assay for the risk to stage 3 T1D. The enhanced analytical sensitivities of ADAP translate to higher identification rates in stage 1 or stage 2 individuals who eventually progress to clinical T1D. The assay also achieved earlier identification of stage 1 or 2 T1D. These favorable clinical performances, together with the low sample consumption and multiplex capability, render the ADAP assay a potentially useful tool for large-scale testing of stage 1 or stage 2 T1D in the general population.

## Data availability statement

The original contributions presented in the study are included in the article/**Supplementary Material**. Further inquiries can be directed to the corresponding author.

## Ethics statement

The studies involving humans were approved by the Western IRB at Enable Biosciences using de-identified specimens. The specimens were sourced from the NIDDK biorepository in the DPT-1 study. The DPT-1 study protocol was approved by the institutional review boards at all participating locations across the U.S. and Canada, including 91 sites in the study. The studies were conducted in accordance with the local legislation and institutional requirements. Written informed consent for participation in this study was provided by the participants’ legal guardians or next of kin.

## Author contributions

DT: Investigation, Methodology, Data curation, Validation, Writing – review & editing. BH: Writing – review & editing, Data curation, Investigation, Validation. FJC: Writing – review & editing, Data curation, Investigation, Methodology, Project administration. DS: Writing – review & editing. PR: Writing – review & editing. C-tT: Investigation, Methodology, Conceptualization, Formal analysis, Funding acquisition, Supervision, Writing – original draft.
